# Hepatitis C Virus and Vaccine Development

**Published:** 2014

**Authors:** Malihe Naderi, Naghmeh Gholipour, Mohammad Reza Zolfaghari, Maryam Moradi Binabaj, Ahmad Yegane Moghadam, Gholamreza Motalleb

**Affiliations:** 1 *Department of Microbiology, Qom branch, Islamic Azad University, Qom 37185-364, Iran.*; 2 *National Institute of Genetic Engineering and Biotechnology, Department of Molecular Genetics, Tehran, Iran.*; 3 *Department of Clinical Biochemistry, Faculty of Medicine, Golestan University of Medical Sciences, Golestan, Iran.*; 4 *Department of Otolaryngology, Kashan University of Medical Sciences, Kashan, Iran.*; 5 *Department of Biology, Faculty of Science, University of Zabol, Zabol, Iran.*

**Keywords:** HCV, DNA vaccine, IFN- α, cellular immune response

## Abstract

The prevalence of Hepatitis C virus (HCV) is approximately 3% around the world. This virus causes chronic hepatitis, liver cirrhosis and hepatocellular carcinoma. The effectiveness of interferon-α and ribavirin therapy is about 50% and is associated with significant toxicity and cost. Hence, generating new vaccines or drugs is an obligation. However, there is no vaccine available for clinical use. DNA vaccines have some advantages such as producing feasibility and generating intensive cellular and humoral immune responses. Activation and improvement of natural immune defense mechanisms is a necessity for the development of an effective HCV vaccine. This article discusses the current status of therapies for hepatitis C, the promising new therapies and the experimental strategies to develop an HCV vaccine.

Hepatitis C virus (HCV) is a positive-sense single-stranded RNA virus of the Flaviviridae family (1). Despite the importance of this virus as a human pathogen, its effect on human health has recently been realized (1). Hepatitis C virus (HCV) can cause chronic liver disease that ultimately leads to hepatocellular carcinoma (2). Contrary to increasing medical and surgical advances in the treatment of HCV-related disease, the biological characteristics and varying immun-osuppressive mechanism of HCV has not yet been completely clarified (3). The HCV infection treatment is difficult and costly because of persistent nature of the virus. Therefore, most infected patients do not receive treatment (4). With respect to high infection incidence, lack of efficient therapy and the current treatment expense of chronic HCV, it seems necessary to use specific strategies to develop a novel immunotherapy (5). On the other hand, the development of new drugs and vaccines can be pursued on a strong funda-mental established procedures and long - term experience (6). However, toxicity is very important in any experimental therapeutic agent (7). The chronic stage of infection is mainly related to HCV pathogenicity, hence there is a need to improve the ability of a vaccine approach to manage or treat this infection (5). Combination of pegylated interferon (IFN) and ribavirin is the common treatment for HCV. Since this regimen is costly, extended, have severe side- effects and is not efficient sometimes (8). On the other hand, recently researchers have shown that tamoxifen suppressed HCV genome replication in a dose dependent manner (9). However, there are crucial issues concerning generating HCV vaccines that will have the ability to prevent and/ or treat this infection (10). In this review, we will first demonstrate the current understanding of HCV infection, pathogenicity and therapy methods and then focus our attention on the significant degree of viral diversity which complicates vaccine development.

**Molecular analysis of the HCV genome**

The HCV genome is a single- stranded RNA which contains a large open reading frame of 9, 030 to 9, 099 bp that could encode a polyprotein of 3, 010 to 3, 033 amino acids (10-12). Polyprotein cleavage and separate protein production occurs at two sites via host signal peptidase in the structural region and HCV-encoded proteases in the non-structural (NS) region. The structural region is composed of the core protein and two envelope proteins (E1 and E2) (11). The defined and hypothetical properties of these genomic proteins are shown in Table 1.

The HCV core protein consists of the first structural protein at the amino end of the polyprotein, and performs several activities (13-14). During maturation, core protein undergoes two sequential membrane- dependent slicing and two types of core protein (amino acids 1 to C173 and amino acids 1 to C191) are produced. These core protein products have cytoplasmic localization. But in the absence of C191, C173 is capable of translocation into the nucleus (15). E1 and E2 glycoproteins encoded by HCV, are hypothesized to be essential in viral envelope formation (16). Previous studies have demonstrated that these structural proteins stimulate the production of neutralizing antibodies and they might also serve as future vaccine candidates (17, 18). These glycoproteins are supposed to play crucial roles in viral entry by binding to the receptor present on the host and in the virus– host immune interactions (19, 20). The NS2 protein is a transmembrane protein anchored to the endoplasmic reticulum with its carboxy terminus while its amino terminus is located in the cytosol (17). Immunoprecipitation investigations (21) revealed that NS2 is closely related to the structural proteins, but the molecular mechanism of action of this protein is poorly understood (3). The HCV non- structural 3 protein (NS3) is a protein of 70 kDa with three known catalytic activities consisting of a serine protease at its N terminus and protease and helicase activities at its C terminus (22). Previous studies showed that NS3 or its fragment may inhibit phosphorylation mediated by cAMP- dependent protein kinase, and interact with or affect the host cell functions (23). The gene product can inhibit the antiviral immune system and is necessary in the life cycle of HCV (24). NS3 revealed novel features and has been one of hot spots in recent researches. Some reports have indicated NS3 specific CD4^+^and CD8^+^ T- cell responses in recovered patients (25). The NS3 serine protease divides NS4 in two parts. The first part, NS4A, is a protease cofactor forming a stable complex with NS3, and a central 12-amino acid peptide has been reported to be important for cofactor activity. The mechanisms by which it enhances NS3 protease activity are not yet known (24, 29, 30) but interaction between NS4A and the extreme N-terminus of NS3 has been reported (31). The function of the last part, NS4b, is not clearly understood but it has been assumed to interact with the HCV replication complex (3). The NS5 is cleaved to NS5A and NS5B (31). Further evidence indicated that NS5A may affect HCV resistance to IFN treatment and inhibition of IFN induced protein kinase. NS5B possesses RNA- dependent RNA polymerase (RdRp) activity that is necessary for HCV replication, and therefore the function of NS5b in HCV has been speculated to be the viral polymerase (3).

**Table 1:** Functions of HCV genomic proteins

**Protein**	**Hepatitis C virus gene function **
NS4B	Replication complex
NS3	Helicase activity
NS5B	Formation of replication complex
p7	Processing of polyprotein, viral assembly
NS2/ NS3	Protease activity
NS3/NS4A	Serine protease activity
E1 and E2	Glycoproteins of envelope

**Infection**

For assessing the effectiveness of antiviral therapeutic approaches and the management of new methods, it is necessary to improve our vision about virus nature (3). The study of HCV replication is difficult because of the absence of permissive culture systems but some studies have generated virus replication using systems from hepatic tissue and peripheral blood mononuclear cells (3). The mechanism of hepatocyte destruction in hepatitis C has been found to be cell- mediated immunity. HCV evades host antibody- mediated neutralization through high variability of its genomic RNA (32). When the virus enters hepatocyte cells via receptor mediated endocytosis, then its replication is initiated and hepatocyte destruction starts by subsequent host’s immune response (33). Because of complicated functions of HCV genomic proteins, the virus is able to evade the natural interferon- mediated clearance (15). HCV infection has been linked to expansion of B lymphocytes in diseases such as mixed- type II cryoglobulinemia (32) and non- Hodgkin’s lymphoma (33). Studies showed that CD81-derived signal in B cells can mediate B cell activation and proliferation (34). HCV through its core protein and NS3 induce production of nitric oxide that causes DNA damage and mutations which play an important role in oncogenesis in HCC (35).

**Fig. 1:**
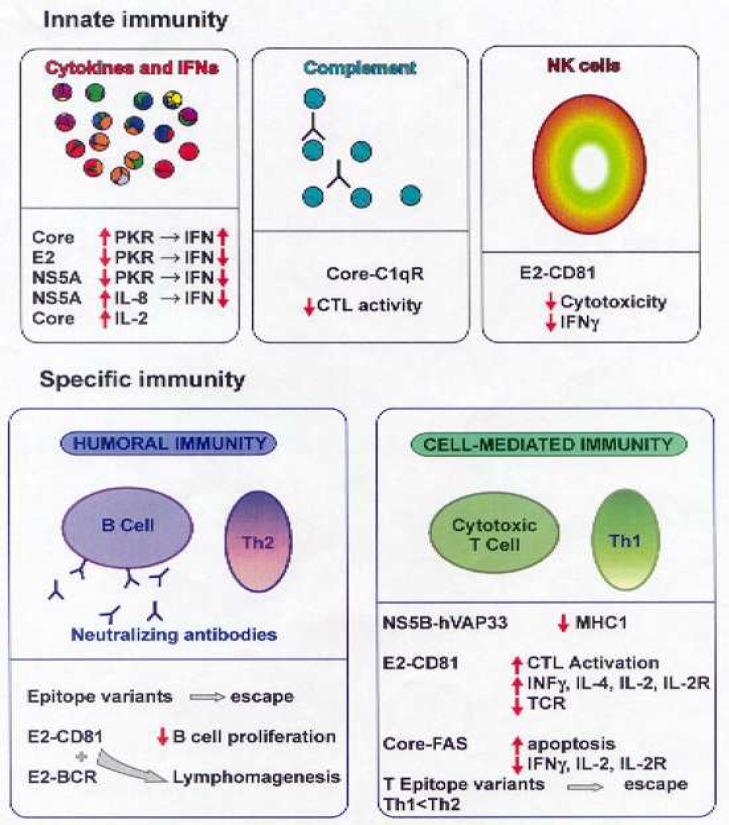
HCV versus the immune system (47). We kindly acknowledge the Indian academy of sciences for permission to reprint Figure 2 in: Pavio N, Lai MMC. The hepatitis C virus persistence: how to evade the immune system . J Biosci 2003; 28(3):287-304

**Fig. 2.**
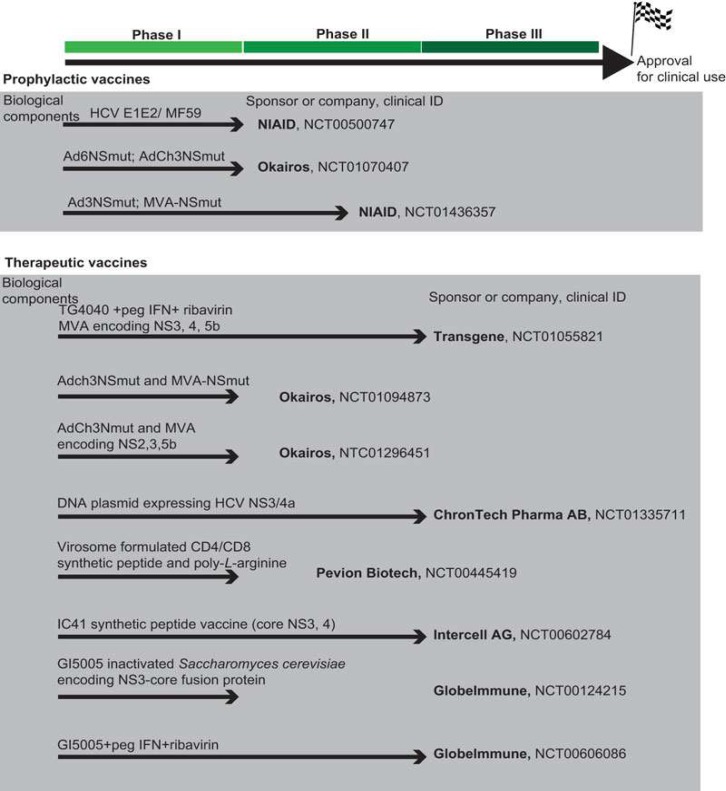
Potential HCV vaccines in clinical phase development. The vaccines are based on either prophylactic or therapeutic usage in phase I, phase I/ II or phase II development (no HCV- specific vaccine has reached phase III development yet). The biological component(s) of the vaccine is listed on top of the arrow. Sponsor or company conducting the trial is listed at the end of arrow along with clinical ID number (http :// www. clinicaltrials.gov) (48). The article has been distributed under a Creative Commons license (Attribution 3.0 Unported (CC BY 3.0). The authors and the Nature Publishing Group are fully acknowledged

**Anti-HCV-specific responses**

An important defense mechanism in viral infections especially in non- cytopathic infections is the histocompatibility complex (MHC) class I-restricted cytotoxic T lymphocyte (CTL) response (36). Virus elimination caused by CTL- mediated lysis of infected cells is a major component of virus clearance and if incomplete, leads to viral continuance and chronic tissue damage (36). Studies showed that cooperative cellular immune responses caused by CD8^+^ and CD4^+^T Lympho-cytes have a key role in managing HCV infection (37, 38). Viral infection induces cellular immune response including non- specific mechanisms, like IFN release and natural killer cell activity, and antigen specific mechanisms like cytotoxic T lymphocytes and inflammatory cytokine release (39) (figure 1). Alpha and beta interferons play a crucial role in treatment of HCV infection and their effectiveness has also been confirmed (40). Previous investigations have revealed early, strong and multi- specific T cell responses to epitopes of HCV proteins correlated with the elimination of viremia and inhibiting viral evade (28). The relationship between vigorous T cell proliferative responses against HCV core, E2, NS3, NS4 and NS5 proteins with self- limited infection is illustrated (41). The key role of HCV-specific T cell response to distinguish the consequences of primary HCV infection is identified (6). The supposed mechanisms of T cell response involves: a) identification of short viral peptides adjoining the groove of MHC molecules by T cells (but evasion of T cell cannot describe the reduced responses in chronic infection); b) decrease in antigen- specific T cell frequency and function because of chronic antigen provocation;c) immune cell subgroup modulation (6). There is a debate about the role of particular antibodies against HCV on management of the infection. Important candidates for virus neutralization are envelope antibodies, but their presence in chronically infected patients and animal studies discuss effective humoral virus neutrali-zation under *in vivo* condition (42, 43). Animal models have played a critical role in establishing basic paradigms in this important studies because they provide an *in vivo* milieu that cannot be reproduced *in vitro*. As novel immunotherapies and cancer vaccines have been developed, animal models have also played an important role in pre-clinical testing for therapeu-tic efficacy (9). Eventually, there is an association between antibody- mediated immune pressure and evolvement of viral evasion mutants within infection (44). Instant induction of neutralizing antibodies in the first stage of infection has a key role in viral elimination (45). Hence, vigorous cellular immu-ne response and induction of neutralizing antibodies are probably essen-tial for prevention of acute infection, viral clearance and effective therapy for chronic HCV infection (46).

**Approaches to HCV vaccinedevelopment**

There are two major approaches to HCV for vaccine development. One is prophylactic and the other is therapeutic vaccines for clinical use (figure 2).

The efficiency of pegylated IFN-alpha plus ribavirin for HCV infection therapy is not always satisfying because it cannot evoke prolonged response in all patients (15). The side effects of this treatment strategy are producing anti- thyroid autoantibodies associated with thyroid dysfunction caused by IFN-α and hematological dyscrasias and considerable hemolytic anemia induced by ribavarin (15, 49). A promising vaccine candidate must be capable of inducing vigorous and prolonged immune responses and having the ability to maintaining protection toward other variety of the same pathogen (50). As well as it should trigger initiation of wide cellular and humoral immune responses against various viral proteins (28). Since HCV has special properties including high replication rate and the error-prone polymerase, development of a vaccine against HCV infection is still a significant obstacle (15). The extent of HCV replication provides ample opportunity for the introduction of mutations into the viral population within an infected individual (15). Heterogeneity and the genetic variability of HCV potentially act as a significant barrier to generate HCV vaccine (51). An effective HCV vaccine would be based on two or several immunogens, might contain various epitopes (52). Moreover, provocating intense and cross- reactive antiviral antibodies and multi- specific cellular immune response are essential for an efficient HCV vaccine (28). New HCV vaccine strategies, for instance, DNA and vector- based vaccines, peptide and recombinant proteins are currently underway in phase I/ II human clinical trials. Some of these vaccines provide an acceptable antiviral immunity in healthy volunteers and infected patients (53). Investigations for the improvement of DNA vaccines against viral and bacterial pathogens showed protection and prolonged immunity (50).

**Recombinant viral vaccine vectors**

Most of the DNA-based vaccine research against various HCV proteins targeted either the humoral or cellular immune responses. However, more powerful vectors should be designed to generate both strong humoral and cellular immune responses against multiple epitopes within the structural and NS proteins. Recombinant viruses are efficient vehicle for DNA release that may cause a high level of recombinant protein expression in host cells (54, 55). Different recombinant viral vectors include adenovirus, vaccinia virus and canarypox virus.

**Peptide vaccines**

Class I MHC molecules exist almost in all cell types and present only intracellulary generated peptide fragments to CD8^+^T cytotoxic cells while class II MHC molecules exist on antigen presenting cells and present antigenic peptides to T helper cells.

Thus, peptide vaccines under this principle make use of small peptides present in the extracellular milieu and can bind directly to MHC class I or II molecules without undertaking the antigen processing ways. Accordingly, chemically synthesized peptides that are potent immunogenic antigens are being pursued as vaccine candidates for HCV (55).

**Recombinant protein subunit vaccines**

A subunit vaccine containing recombinant HCV proteins can save from harm from infection or chronic infection by different HCV genotypes. The first effort to develop an HCV vaccine was directed toward generating a recombinant protein subunit vaccine. Since it has been shown for several flaviviruses that antibodies to the envelope protein can supply protection, recombinant HCV E1 and E2 proteins were used in early vaccination studies from Chiron (55, 56).

**DNA vaccine**

One of the latest versions of vaccine is DNA- based immunization method (55). DNA vaccines have shown superiority effects compared with conventional vaccines, such as recombinant protein- based vaccines and live weakened viruses (5). DNA immunization advantages include feasibility of production, DNA manipulating simplicity and immune responses resulting primarily from different origins such as T helper cell and CTL, and antibody responses (55). Also, DNA vaccines are suitable for sequential vaccinations since their function is not influenced by pre-existing antibody titers to the vector (52). HCV is a so high variant virus that it is difficult to develop HCV vaccine (57). NS3 gene is partially conserved and by inducing specific T cell responses plays a major role in HCV clearance making it a suitable candidate for T cell based vaccines, since most researchers have concentrated on the specific CTL response stimulated by C and NS3 region proteins and protective antibodies induced by HCV E proteins (27, 57). HCV core should be destroyed under the influence of immune response induced by a specific vaccine. It is a potent immunogen with anti-core immune response that arise during the early stage of infection (58). The HCV core protein might seem the obvious candidate for a therapeutic T-cell vaccine, since this is the most highly conserved region of the translated HCV genome both within and between different HCV genotypes. However, studies have shown that the core protein can interfere with innate and adaptive anti-HCV immune responses (59, 60). DNA-based vaccines are inferior to the traditional vaccines such as subunit vaccines since the intensity of the immune responses induced by DNA vaccines has been relatively weak, therefore attempts are directed towards the development of new technique like co-delivery of novel cytokine IL-2, IL-7, IL-12, IL-15 and IL-18 adjuvants for circumventing this restriction (61).

HCV identification is indeed the most considerable recent development in viral disease. Because of the clinical significance of the disease, researches into the development of new therapeutic strategies are accentuated on the study of molecular properties of the virus. An efficient HCV vaccine should stimulate the different aspects of the immune response such as broad humoral, T helper and CTL responses.

Since the HCV genome demonstrates high heterogeneity and mutagenicity, generating prophylactic or therapeutic vaccine for HCV is still an unsolved problem. Previous studies illustrated that the cellular immune responses might be essential for an efficient vaccine. New vaccine candidates, including DNA, peptide, recombinant protein and vector-based vaccines have been shown to have many advantages and lately have entered onto phase I/II human clinical trials. Some of these strategies provide an acceptable antiviral immunity in healthy volunteers and infected patients, but the challenge is to examining their effectiveness in infected or at- risk populations.

## Conflict of interests

The authors declared no conflict of interests.
